# Pneumatosis cystoides intestinalis with colonic perforation: A case report and literature review

**DOI:** 10.1097/MD.0000000000046904

**Published:** 2025-12-26

**Authors:** Jihua Cao, Daigui Zi, Min Chen, Ziyue Xiang

**Affiliations:** a Department of Hepatobiliary Surgery, The First College of Clinical Medical Science, China Three Gorges University, Yichang Central People’s Hospital, Yichang City, China; b Department of General Surgery, Xingshan County People’s Hospital, Yichang City, China; c Department of Operating Room, The First College of Clinical Medical Science, China Three Gorges University, Yichang Central People’s Hospital, Yichang City, China.

**Keywords:** colonic perforation, pneumatosis cystoides intestinalis, radiological manifestations, surgery, treatment

## Abstract

**Rationale:**

Pneumatosis cystoides intestinalis (PCI) is an uncommon complaint that can produce free gas below the diaphragm, mimicking gastrointestinal perforation, which can easily lead to misdiagnosis. Although prognosis is generally good in most cases, complications can pose a threat to life and may require surgical intervention. Currently, clear surgical guidelines for PCI complicated by colonic perforation are lacking.

**Patient concerns and diagnoses:**

We present the case of a 57-year-old male patient with pneumatosis cystoides intestinalis complicated by colonic perforation. The patient was admitted because of persistent upper abdominal pain for 3 days, which worsened over 5 hours. The client suffered from a history of chronic obstructive pulmonary disease, pulmonary bullae, and heart failure.

**Interventions and outcomes:**

Emergency surgery was performed, during which severe abdominal contamination, significant colon dilation, cystic bubbles in the intestinal wall, and perforation of the splenic flexure of the transverse colon were observed. Consequently, the doctors performed partial colectomy and colostomy. Postoperatively, due to recurrent fever symptoms, he was transferred to a higher-level hospital for further treatment and was ultimately discharged successfully.

**Lessons:**

PCI combined with colonic perforation should be regarded as a routine differential diagnosis for urgent abdominal pain. Although conservative treatment is the preferred option in most cases, timely surgical intervention should be considered in the presence of complications, hemodynamic instability, signs of peritonitis, and significant abnormal laboratory and imaging findings. Based on the current evidence, partial colectomy with colostomy is recommended for patients undergoing PCI combined with colonic perforation.

## 1. Introduction

Pneumatosis cystoides intestinalis (PCI) is an uncommon disorder characterized by the formation of air-filled vesicles in the submucosal and/or subserosal layers of intestinal walls.^[1]^ This disease typically occurs in the colon and small bowel, with an incidence of approximately 0.03%.^[2]^ The absence of specific clinical symptoms makes colonic PCI easy to overlook or misdiagnose as colonic PCI. Although the prognosis is generally good for most patients, some may develop severe complications.

Reports on cases of colonic PCI with colonic perforation are relatively rare, and there is no consensus regarding surgical approaches. This report presents an unusual case of colonic PCI combined with transverse colon perforation. A detailed summary of the surgical methods for such patients was conducted through a literature review, aiming to provide a reference for the formulation of future related guidelines.

## 2. Case report

A 57-year-old male was brought to the doctor because of persistent epigastric pain lasting for 3 days that worsened over 5 hours. He had a history of pulmonary bullaechronic obstructive pulmonary disease (COPD) and heart failure. During physical examination, diminished breath sounds in both lungs were noted, along with wheezing and moist rales. The abdomen was tense, with tenderness and rebound tenderness (+), and bowel sounds were diminished.

**Figure 1.**
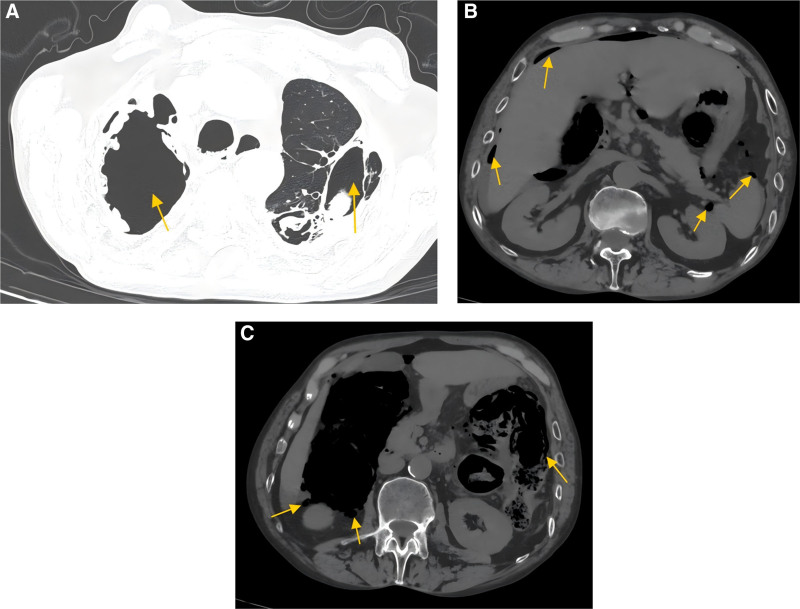
The CT scans of the lung and abdomen. (A) Bilateral upper lobe multiple pulmonary bullae; (B) Free intraperitoneal gas; (C) Multiple pneumatosis of the colonic wall. CT = computed tomography.

Biochemical examination showed hypoproteinemia, normal white blood cell count, hemoglobin level of 110 g/L, and procalcitonin level of 1.7 ng/mL. Computed tomography (CT) revealed pulmonary bullae, abdominal free air, ascites, pelvic effusion, and inflammatory changes in the mesentery (Fig. 1).

**Figure 2.**
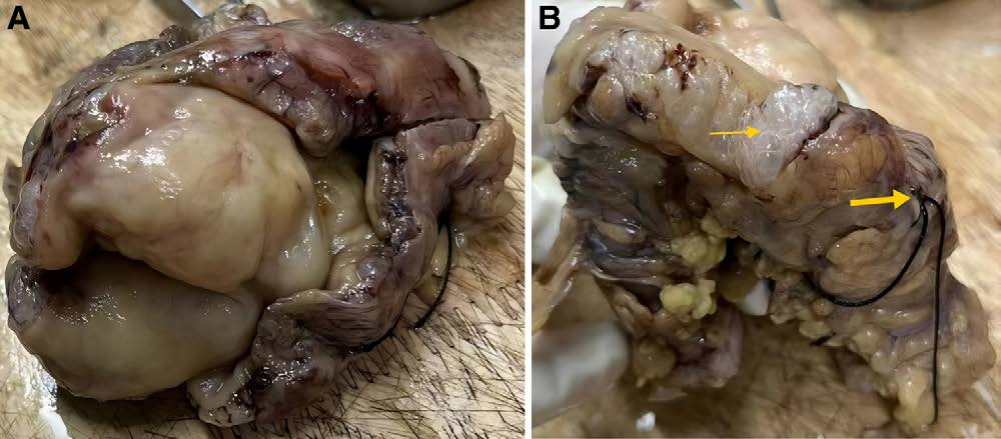
Resected colon specimen. (A) Significant mucosal edema of the intestine, with disappearance of the folds. (B) Submucosal multiple transparent vesicles (thin yellow arrow), colonic perforation (thick yellow arrow).

**Figure 3.**
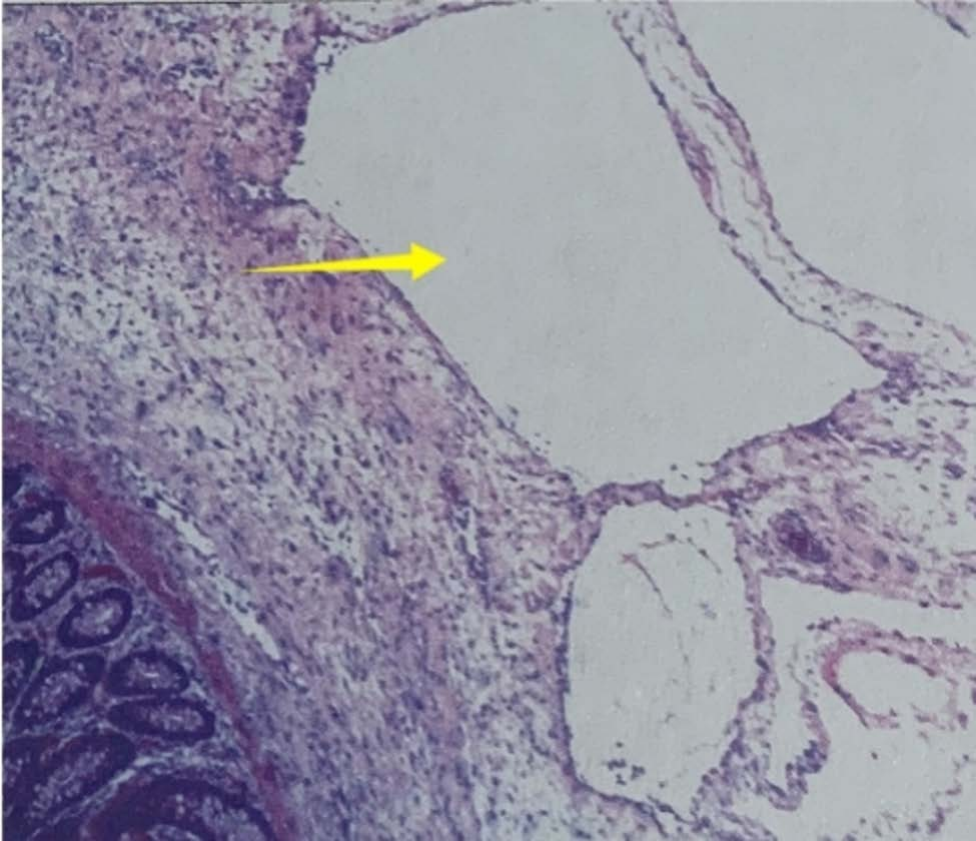
Under the microscope, the colon section shows chronic inflammatory changes and numerous gas-filled vesicular structures positioned in the submucosal layer.

Given the urgency of gastrointestinal perforation, emergency surgical exploration was performed. During the operation, severe contamination of the abdominal cavity was observed with significant colonic dilation, and multiple cystic bubbles were found on the colonic wall. The perforation site is located in the splenic flexure region of the transverse colon. Consequently, a partial colectomy and transverse colostomy were performed. The patient was transferred to the ICU for treatment and subsequently transferred to a higher-level hospital for further treatment owing to persistent fever symptoms. He received negative pressure drainage treatment with drainage tube in the higher-level hospital, and upgraded antibiotics to meropenem. After half a month of treatment, he was discharged after improvement. Gross examination of the specimen revealed significant edema of the intestinal mucosa, disappearance of the plicae, a loose submucosal layer similar to a sponge, and visible diffuse transparent vesicles (Fig. 2). Pathological examination indicated PCI with perforation (Fig. 3).

## 3. Discussion and conclusion

PCI is a relatively uncommon disorder that was first identified by Duvernoy during an autopsy. It can be located anywhere in the digestive channel but occurs most frequently in the small intestine in 36.4% of cases, while the colorectum is affected in 29.7% of patients.^[3]^ Depending on etiology, PCI has been classified into primary (15%) and secondary (85%) types.^[4]^ Currently, >60 causative factors are associated with secondary infections.

PCI is usually symptomless or exhibits nonspecific symptoms such as bloating, epigastric pain, diarrhea, or constipation.^[1]^ When severe, bowel obstruction, torsion, intussusception, necrosis, and perforation can arise, affecting approximately 3% of cases.^[5]^

However, the exact pathogenesis of PCI remains unknown. Several theories have been proposed, including mechanical, pulmonary, bacterial, chemical, and nutritional factors. Mechanical theory suggests that increased pressure within the intestinal lumen may cause rupture of the mucosa, allowing gas to penetrate the intestinal wall. The pulmonary theory posits that alveolar rupture, triggered by respiratory diseases, may allow gas to enter the intestinal wall through large blood vessels. The bacterial theory states that gas-producing bacteria penetrate the mucosal barrier and produce gas within the intestinal wall, whereas the chemical or nutritional theory holds that an increase in acidic substances promotes bacterial fermentation, resulting in the production of large amounts of gas, which may increase the risk of PCI.^[6]^ The patient had previously suffered from COPD and pulmonary bullae and presented with symptoms of chronic cough. Therefore, we believe that pulmonary theory may have played a key role in this case.

The diagnosis of PCI is often serendipitous, especially in asymptomatic patients, and it is usually discovered incidentally during imaging examinations for other reasons or during surgery. The diagnosis of the disease can be achieved with the help of X-ray imaging or CT scans, and it is generally accepted that CT is the imaging technique with the highest sensitivity.^[7]^ On X-ray imaging, PCI usually appears as bubbly or linear gas shadows around the intestinal wall, which may be distributed along the mesentery or bowel. On a CT scan, gas cystic cavities within the bowel wall can be more clearly visualized and, as demonstrated in this patient, these cavities may appear round, oval, or irregularly shaped, with varying sizes and smooth or irregular edges. Additionally, CT may also show bowel wall thickening, edema, mesenteric inflammatory changes, and other associated changes, such as abdominal free air and abdominopelvic effusion, and enteroscopy also plays an important role in PCI diagnosis.

Misdiagnosis of PCI is not uncommon, especially when free gas is detected in the abdominal cavity on imaging, which is often assumed to be perforation of a cavity organ, prompting the surgeon to perform an exploratory laparotomy.^[8,9]^ Nevertheless, free gas may originate from the disintegration of gas-filled vesicles into the peritoneal cavity rather than from an actual perforation. Statistically, intraperitoneal free gas is observed in approximately 2% of colonic PCI cases compared to 15% in the small bowel.^[10]^ The clinical and radiological presentation of PCI can mimic several other abdominal conditions, making differential diagnosis crucial. Key conditions to consider include: Gastrointestinal perforation due to peptic ulcer disease, diverticulitis, or appendicitis, which typically presents with acute abdominal pain, guarding, rebound tenderness, and free air on imaging; Bowel ischemia or necrosis, which may present with severe pain, lactic acidosis, and signs of sepsis. CT may show pneumatosis but often with portal venous gas and bowel wall thickening; Inflammatory bowel disease (e.g., Crohn disease or ulcerative colitis) that can cause transmural inflammation and pneumatosis in rare cases; Infectious colitis such as Clostridium difficile infection, which can lead to pneumatosis and perforation in severe cases; Iatrogenic causes including endoscopy-related injury or chemotherapy-induced mucosal damage. Differentiating PCI from these conditions requires a combination of clinical history, physical examination, laboratory findings (e.g., lactate, inflammatory markers), and detailed imaging review. The presence of cystic or linear gas patterns confined to the bowel wall, without features of transmural inflammation or portal gas, may favor PCI.

True gastrointestinal perforation is often accompanied by peritonitis, changes in laboratory tests (such as metabolic acidosis, lactate levels >2 mmol/L, and abnormal elevation of infection indicators), and even hemodynamic instability. Therefore, in the diagnostic process, the patient’s vital signs, clinical manifestations, laboratory tests, and imaging features must be considered in a meticulous analysis to avoid misdiagnosis. This patient was admitted to the hospital with acute abdominal pain, although his vital signs were temporarily stable and his leukocytes were normal. Elevated calcitonin, free gas under the diaphragm detected on CT scan, bubble-like changes in the wall of the colon, and inflammatory manifestations of the abdominal cavity, combined with his peritonitis signs, led to the diagnosis of PCI combined with gastrointestinal perforation on comprehensive analysis.

Treatments for PCI consist of 2 main modalities: conservative treatment and surgery. The specific plan should be based on the symptoms and complications, combined with the potential causes of comprehensive evaluation. In patients with no symptoms or mild symptoms, intestinal air sacs can be absorbed spontaneously without special intervention; In patients with obvious symptoms but no serious complications, conservative treatment strategy is recommended. Conservative treatment measures include: oxygen therapy (high-flow or hyperbaric oxygen) to reduce intracavitary gas pressure and accelerate cysts absorption; antibiotic therapy (e.g., metronidazole) targeting gas-producing bacterial; treatment of underlying conditions such as COPD, connective tissue diseases, or autoimmune disorders; dietary adjustments to reduce intestinal gas production.^[11]^ During conservative treatment, close monitoring of changes in the condition and regular reviews are required to assess the progress of the disease and to adjust the treatment plan on time. The condition of most patients can be improved with conservative treatment. However, in cases of complicated PCI—such as bowel perforation, peritonitis, bowel obstruction, intussusception, or signs of sepsis—surgical intervention is indicated. The surgical approaches include simple bowel perforation repair, bowel resection with primary anastomosis, and bowel resection with stoma. Individualized decisions should be made by considering factors such as the degree of peritoneal contamination and hemodynamic stability. For cases of diagnostic uncertainty, laparoscopic exploration may be considered to avoid unnecessary laparotomy, but there is currently a lack of clear surgical guidelines in this field.

Reviewing the literature published over the past decade^[12–16]^ (Table 1), a total of 5 cases of colonic perforation combined with PCI have been documented. Of these cases, 5 patients underwent colectomy, with an additional enterostomy in 3, and only 1 patient opted for repair of the colonic perforation. All the patients showed a favorable postoperative prognosis. Our surgical approach is consistent with the techniques used in most hospitals. Therefore, we recommend that partial colectomy with colostomy should be considered in patients undergoing PCI combined with colonic perforation. In addition, simple repair and drainage of bowel perforations may be considered for patients with lesser abdominal contamination and smaller perforation diameters. However, further studies are required to validate this approach.

**Table 1:** Literature reports on PCI combined with colonic perforation.

References	Gender	Age	Location	Treatment
Yuan 2022^[12]^	Man	90	Sigmoid colon	Partial colectomy + colostomy
Habeb 2025^[13]^	Man	60	Sigmoid colon	Colon perforation repair
Tirumanisetty 2019^[14]^	Woman	75	Ascending colon	Partial colectomy + enterostomy
Guan 2018^[15]^	Woman	78	Ascending colon	Partial colectomy + enterostomy
Gassend 2016^[16]^	Unknown	72	Transverse colon	Partial colectomy

PCI = pneumatosis cystoides intestinalis.

Overall, PCI combined with colonic perforation, although uncommon, should never be taken lightly. In imaging examinations, the presence of free gas below the diaphragm does not specifically indicate gastrointestinal perforation. Although conservative treatment is the preferred option in most cases, timely surgical intervention should be considered in the presence of complications, hemodynamic instability, signs of peritonitis, and significant abnormal laboratory and imaging findings. Based on the current evidence, partial colectomy with colostomy is recommended for patients undergoing PCI combined with colonic perforation.

## Author contributions

**Conceptualization:** Ziyue Xiang.

**Resources:** Daigui Zi.

**Supervision:** Min Chen, Ziyue Xiang.

**Validation:** Daigui Zi.

**Visualization:** Ziyue Xiang.

**Writing – original draft:** Jihua Cao.

**Writing – review & editing:** Jihua Cao.
